# Supplementary material for: Development of a multivariate prediction model of intensive care unit transfer or death: A French prospective cohort study of hospitalized COVID-19 patients
Source: PLoS One. 2020 Oct 19;15(10):e0240711. doi: 10.1371/journal.pone.0240711 (PMC7571674; doi:10.1371/journal.pone.0240711)
Supplement: S1 File — (DOCX) [file pone.0240711.s001.docx]

**Supplementary material**

1. **Description of the external validation cohort**

**Table S1. Description of the external validation cohort**

| **Variable** | **Development sample** | **External cohort** |
| --- | --- | --- |
| Total | 152 | 131 |
| Male patients | 91 (59.9) | 79 (60.8) |
| NA, nb. | 0 | 1 |
| Age at admission (years) | 77 (60;83) [20 ; 99] | 60 (49;74) [17 ; 96] |
| Baseline respiratory status |  |  |
| No oxygen | 44 (29) | 32 (24) |
| Oxygen by nsal prongs or mask | 89 (59) | 76 (58) |
| High flow oxygen or NIV | 19 (12) | 23 (18) |
| Lymphocytes (/mm3) | 805 (600;1148) | 1040 (842;1280) |
| C-Reactive protein (mg/L) | 74.5 (30.9;135.1) | 88.0 (42.0;160.5) |
| ICU transfer or death within 14 days | 47 (32.0)* | 36 (29.8) |
| NIV, MV, or death, at day 14 | 40 (27.4)‡ | 36 (27.5) |
| Discharged alive within 14 days | 84 (57.5)‡ | 75 (57.3) |

*Data available for 147 patients; ‡Data available for 146 patients

1. **Statistical aspects**

Multivariable models were estimated using logistic regression with maximum likelihood. A sensitivity analysis using the Fine and Gray model accounting for the time to the event (ICU transfer or death between day 1 and 14, with discharge alive without ICU stay as a competing event; discharge alive between day 1 and 14, with hospital death as a competing event) yielded similar results on the association of the variables with the outcomes distribution.

Quantitative predictors were considered as continuous variables (except for age, which was dichotomized, above or below 60 y.o., based on clinical input) and qualitative as binary or dummy variables, for model development. Log-linearity of quantitative predictors was evaluated with comparison to a restricted cubic spline modeling. The set of predictors (age, respiratory status at baseline summarized using WHO scale, CRP level, lymphocytes count) was defined after checking for redundancy among candidate predictors based on clinical expertise, and accounting for an acceptable number of degrees of freedom of 4, given the limiting number of events. We considered that variables which could not be easily and routinely collected at baseline were not relevant to elaborate a routine pronostic model and score; therefore, candidate variables with more than 20% missing data were not considered for the pronostic model. There was no evidence of multicollinearity among the selected predictors. No statistical-based variable selection was performed. Multivariable logistic regression models were estimated on complete cases samples (n=147 for the main endpoint ICU transfer or death within 14 days, n=146).

First, internal validation via 1000 bootstrap resampling was performed for each outcome. Estimates on the development sample and performances (with internal and external validation) are reported in the table S2 below, and calibration curves in figures S1 and S2.

For rough field risk-assessment, we computed a tentative simplified score based on the linear predictor and the coefficients of the multivariable model predicting the main endpoint (ICU transfer or death within 14 days of admission). Unit points were allocated as follows:

- 1 point for age above 60 y.o.;
- 1 point for WHO scale at 4, 3 points for WHO scale 5;
- CRP level was treated as:
  - 1 point if 10 ≤ CRP ≤ 75 mg/L,
  - 2 points if 75 ≤ CRP ≤ 150 mg/L,
  - 3 points if CRP ≥ 150 mg/L;
- 1 point if lymphocytes count below 800/mm3.

The obtained groups of patients are illustrated in figure 2 of the main manuscript, for the main endpoint, ICU transfer or death within 14 days, in the development sample and the external validation sample.

On the development sample, the score resulted in a discrimination C-index of 0.785, corrected for by internal bootstrap resampling. The score was applied to the external sample (see figure 3, main manuscript), yielding a C-index of 0.774 (see figure S3).

**Table S2. Multivariable models, development sample, with internal validation via 1000 bootstrap resampling**

|  | **ICU transfer or death within 14 days** | |  | **Discharge alive within 14 days** | |  | **VNI, MV or death at day 14** | |
| --- | --- | --- | --- | --- | --- | --- | --- | --- |
|  | **Coefficient (SE)** | **P-value** |  | **Coefficient (SE)** | **P-value** |  | **Coefficient (SE)** | **P-value** |
| Intercept | -6.584 (1.769) |  |  | 6.064 (1.694) |  |  | -8.694 (2.154) |  |
| Age > 60 y.o. | 0.960 (0.510) | 0.060 |  | -1.626 (0.501) | 0.001 |  | 2.514 (0.821) | 0.002 |
| WHO scale | 1.395 (0.423) | 0.001 |  | -1.205 (0.406) | 0.003 |  | 1.430 (0.461) | 0.002 |
| CRP (per 100 mg/L increment) | 0.490 (0.260) | 0.059 |  | -0.708 (0.288) | 0.014 |  | 0.647 (0.294) | 0.028 |
| Lymphocytes count (per 1000/mm3 increment) | -1.035 (0.523) | 0.048 |  | 0.876 (0.445) | 0.049 |  | -0.817 (0.554) | 0.140 |
|  |  |  |  |  |  |  |  |  |
| ***Performance measures*** | *Internal** | *External* |  | *Internal** | *External* |  | *Internal** | *External* |
| *Somers’ Dxy* | 0.572 | 0.575 |  | 0.605 | 0.654 |  | 0.666 | 0.622 |
| *C-index* | 0.786 | 0.787 |  | 0.803 | 0.827 |  | 0.832 | 0.811 |
| *R²* | 0.303 | 0.327 |  | 0.363 | 0.391 |  | 0.385 | 0.306 |
| *Calibration intercept* | -0.055 | -0.105 |  | -0.006 | -0.257 |  | -0.067 | 0.076 |
| *Calibration slope* | 0.885 | 0.885 |  | 0.910 | 0.928 |  | 0.848 | 0.703 |
| *Brier score* | 0.167 | 0.154 |  | 0.181 | 0.170 |  | 0.141 | 0.145 |

* All values corrected for over-optimism by internal validation via 1000-bootstrap resampling.

**Figure S1. Calibration curves for the multivariable models respectively for each endpoint (top left panel: ICU transfer or death within 14 days, top-right panel: discharge alive within 14 days, bottom panel: NIV, ICU or death at day 14), with internal validation by 1000-bootstrap resampling.** Observed event probabilities (y-axis) against model predictions (x-axis) (dotted line: development sample, solid line: corrected using internal validation by bootstrap resampling, dashed line: reference ideal calibration)

**Figure S2. Calibration curves for the multivariable models respectively for each endpoint (top left panel: ICU transfer or death within 14 days, top-right panel: discharge alive within 14 days, bottom panel: NIV, ICU or death at day 14), on the external sample.** Observed event probabilities (y-axis) against model predictions (x-axis) (solid line: logistic calibration on the external sample, gray line: reference ideal calibration)

**Figure S3. Calibration curves for the simplified score, on the development sample (left panel), and the external sample (right panel), for the main endpoint: ICU transfer if death within 14 days of admission.** Observed event probabilities (y-axis) against model predictions (x-axis) (solid line: logistic calibration, diagonal line: reference ideal calibration)
