# Supplementary material for: Development of a multivariate prediction model of intensive care unit transfer or death: A French prospective cohort study of hospitalized COVID-19 patients
Source: PLoS One. 2020 Oct 19;15(10):e0240711. doi: 10.1371/journal.pone.0240711 (PMC7571674; doi:10.1371/journal.pone.0240711)
Supplement: Attachment — Submitted filename: response to the editor about Data Sharing.docx [file pone.0240711.s002.docx]

Dear Editor ,

I’m sending you this letter in response to your latest inquiry about Data sharing.

As mentioned in our statement, we do not have the right to share the data of our patients due to the restriction of the ethical committee that gave the approval.

I added in the uploaded files the ethical committee approval document that clearly states that we need to abide to the GDPR (General Data Protection Regulation in Europe). Any data that can identify the patients are not allowed to be shared even upon request. We are representing the data of all the patients that were hospitalized during the first weeks of the COVID-19 pandemic in Paris in two specific medical department. Even without their name, the patients can be easily identified by all the other data presented. Hence , sharing our data publically would compromise patient confidentiality and the privacy of the participant.

b) The name of the ethics committee that gave the approval is ‘ comité d’éthique de la recherche , CER- Sorbonne Université ’ . The certificat number is ‘CER-2020-14’

c) Data of our study cannot be shared . In need of confirmation of our statement, the Editor can join Mr Patrick Lefebvre, the referent of the GDPR in the Pitié Salpetriere University Hospital .

Mr Patrick Lefebvre

Email: [Patrick.lefebvre@aphp.fr](mailto:Patrick.lefebvre@aphp.fr)

Tel: 0033631145043

Fax: 0033142162527

**Address:** DSI GH Pitié Salpetriere , 47-83 Bd de l’Hôpital, 75651 Paris cedex 13

We stay at your disposal for any other inquiry.

Yours Truly,

Dr Georgina Maalouf
